# Micro and Nano Raman Lasers

**DOI:** 10.3390/mi12010015

**Published:** 2020-12-25

**Authors:** Luigi Sirleto

**Affiliations:** Institute of Applied Sciences and Intelligent Systems (ISASI), CNR, 80131 Napoli, Italy; luigi.sirleto@cnr.it

Raman lasers (RLs) are a class of optically pumped laser, offering coherent lights at any desired wavelength by a proper choice of the pump wavelength, when both wavelengths are within the transparency region of the gain material and an adequately high nonlinearity and/or optical intensity are provided [1]. RLs are based on Stimulated Raman scattering (SRS); it is a nonlinear optical process governed by the vibrational frequency of the gain material. In contrast to optical parametric oscillators (OPOs), SRS does not require phase matching, but can only provide discrete sets of wavelength shifts [2]. In a RL, an amplifier medium based on Raman gain is used rather than on stimulated emission from excited atoms or ions. The required pumping wavelength can be chosen to minimize absorption, since it does not depend on the electronic structure of the medium. In RLs, with the emission wavelength determined by the pump wavelength, a broad tunability is enabled [1,2]. 

In the past century, silica has been the main material used for long- and short-haul transmission of optical signals, because of its good optical properties and attractive figures of merit. Nowadays, the growing demand in terms of transmission capacity has fulfilled the entire spectral band of the erbium-doped fiber amplifiers (EDFAs). This dramatic increase in bandwidth rules out the use of EDFAs, leaving Raman lasers as the key devices for future amplification requirements [3]. However, in order to answer telecommunications demands, the investigation of new glasses materials with both large Raman gain coefficients and spectral bandwidth is required [4,5]. 

In the past two decades, important accomplishments have been achieved by micro and nano Raman lasers [6], opening new perspectives for the realization of more efficient Raman lasers with ultrasmall sizes. 

The aim of microphotonics is to offer a reliable platform for dense integration. During the past decades, the fast growth of microscales fabrication techniques has enabled the successful demonstration of various types of microphotonics devices, for example, ring resonators and photonic crystals (PhCs). In a microphotonics device, photons are trapped in small volumes close to the diffraction limit for sufficiently long times, so that an increasing of the local field is achieved, which is proportional to the Q value of the resonator and inversely proportional to its volume. Since photons strongly interact with the host material, nonlinear effects are enhanced, and a significant reduction of their power threshold is obtained [7,8]. 

Ultrahigh quality factor (*Q*) optical resonators in silica are a unique platform for developing Raman lasers. In 2002, the first micro Raman laser, realized in silica microspheres with diameters of the order of tens of micrometers and operating in a single-mode as well as in a multimode regime, was implemented [9]. In that laser, a significant pump threshold reduction and a pump-signal conversion higher than 35% were achieved [9].

In 2016, a high-efficiency Raman laser based on Zr-doped silica hybrid microcavities was demonstrated. Ultrahigh-*Q* silica toroidal microcavities were coated with different Zr-doped sol–gels. The Raman gain of the Zr-doped silica showed an increasing dependence on Zr dopant concentration. Unidirectional pump-to-Raman conversion efficiency, exhibiting a marked enhancement up to 47%, was demonstrated [10].

Single-mode Raman microlasers are an attractive option for the development of widely tunable low-threshold single-mode light sources. In [11], a single-mode Raman microlaser, realized in silica microsphere with a diameter of 166 μm and with a Q factor of 2 × 10^7^, was demonstrated. The silica micro Raman laser was tunable in the U-band and beyond (in the 1631–1685 nm range). 

Silicon (Si) photonic devices, fabricated on silicon-on-insulator (SOI) wafers by complementary metal–oxide–semiconductor (CMOS)-compatible processes, have made substantial progress in this decade [12]. Among these devices, compact Si-based lasers are considered to be fundamental for several applications such as opto-electronic integrated circuits and short-distance optical communication. However, due to its indirect bandgap, the resulting radiative efficiency of the electron–hole recombination is low, and optical gain is weak. Consequently, the realization of Si-based lasers using interband transitions has proven to be very difficult, and the use of Stimulated Raman scattering (SRS) is the only option. Si is transparent in the near-infrared optical communication bands, and its Raman gain coefficient is five orders of magnitude larger than silica. In 2005, a Raman laser in silicon waveguide was successfully implemented, enabling continuous-wave (cw) operation at room temperature [13]. A Raman microlaser in cw operation with a threshold of 20 mW and based on a high-Q Si ring resonator was realized too [14]. However, the narrow bandgap (∼1.1 eV) of silicon makes significant the generation of two-photon absorption (TPA) at the telecom band [13,14]. Carriers generated by TPA cause various detrimental effects, which can influence laser performance.

Photonic crystal (PC) nanocavities are highly attractive for implementation of compact Raman Si lasers. In order to confine the pump light and Stokes Raman light, nanocavity-based Raman Si lasers utilize two high-Q resonant modes with an energy separation of about 15.6 THz, which equals the Raman shift of Si. PC nanocavities have a very compact resonator size (about 10 µm) and they are able to achieve high Q values, larger than 10 million. These features enable ultralow thresholds (microwatt or even submicrowatt) Raman lasers and make these devices suitable for the dense integration of Si lasers in Si photonic circuits [15,16]. 

Usually, nanocavities are fabricated by electron-beam (EB) lithography, which provides high accuracy but is a relatively time-consuming method. Recently, mass production of nanocavities with Q values larger than 2.5 million has been achieved by CMOS-compatible processes, including photolithography [17]. A Raman Si laser based on a nanocavity fabricated by argon fluoride (ArF)-based immersion lithography has been demonstrated. Room-temperature cw oscillation was observed above the threshold of 1.8 µW [18]. However, there are still several difficulties in fabricating Raman Si nanocavity lasers by CMOS-compatible processes; one of the most relevant is that the nanocavity has to be fabricated along the [100] crystal direction of Si in order to enhance the Raman gain [19,20]

Diamond is a reasonable material for compact, on-chip Raman lasers over a wide spectrum. A CW low-threshold Raman laser, based on waveguide-integrated diamond racetrack microresonators embedded in silica on a silicon chip, was demonstrated in 2015 [21]. Pumping at telecom wavelengths, a tunable Stokes output over a ∼100 nm bandwidth around 2 µm, with output power greater than 250 µW, was reported. However, the preparation of high-quality diamonds for integrated waveguide fabrication still remains a challenge [21]. 

The recent advancement in lithium-niobite-on-insulator (LNOI) technology is opening up new opportunities in optoelectronics, due to large χ^(2)^ and χ^(3)^ nonlinearities along with the power of dispersion engineering. In [22], the dominant mode for the Raman oscillation was observed in the backward direction for a continuous-wave pump threshold power of 20 mW with a high differential quantum efficiency of 46%. 

Surface-bound molecules have been identified by their Raman vibrational modes, and polarization-dependent Raman gain has been demonstrated. In a surface Raman process, the Raman scattering intensity is dominated by the orientation of the vibrational mode with respect to the polarization of the incident wave at the surface. Therefore, to realize surface SRS it is necessary to establish a high-intensity optical field of a single polarization that is aligned with the molecular vibrational modes. However, in order to realize a surface-constrained Raman laser, a sufficiently high photon population located at a surface to transition from spontaneous to stimulated Raman scattering has to be achieved. By creating an oriented, constrained Si-O-Si monolayer on the surface of integrated silica optical microresonators, the requisite conditions for SSRS have been achieved with low threshold powers (200 µW). Due to the ordered monolayer, the Raman lasing efficiency can be improved from ~5% to over 40% [23].

Liquids can have Raman gain that makes them proper for ultrahigh-Q Raman lasers. High index contrast between the liquid and its air-cladding benefits total internal reflection near the air–liquid interface, while uniquely confining light to overlap almost entirely with the liquid core. In [24], a Raman laser emission from a liquid-walled optofluidic device in the form of a droplet resonator coupled to a tapered fiber was demonstrated. 

Concerning nonlinear nanophotonics devices, we will have a big demand in the near future. Such devices should allow us to control light, with light in a very thin nanoscale layer or in a single nanoparticle of nonlinear material. In principle, in order to control a signal light in a nonlinear optical device, the intensity or phase of light has to be changed by a control signal, thus changing the optical characteristics of the medium. Of course, the stronger the nonlinearity of the material, the shorter the required interaction length L. Since in nanoscale devices the nonlinear effects cannot be enhanced using photon confinement effects, their performances only depend on the nonlinearity of the medium. Therefore, a development of nanostructured materials with large nonlinearities and satisfying also various technological and economical requirements is mandatory. During the past few decades, a significant number of nanomaterials were shown to have notable nonlinear optical properties [25]. One of the most promising materials for light emission applications in nanophotonics is silicon nanocrystals (Si-nc) [26,27,28,29]. Concerning SRS in silicon nanocrystals (Si-nc), a giant Raman gain was measured at the wavelength of interest for telecommunications. An impressive enhancement of the Raman gain in Si-nc up to four orders of magnitude, compared with bulk silicon, as a function of Si concentration, was observed [28,29]. Since the SRS effect in Si-nc is about 10^4^ times larger with respect to silicon, in principle a Raman laser with typical dimensions of a few micrometers could be obtained. Therefore, all the advantages of combining optical and electronic functions on a single chip could be experienced. 

In [30], the first theoretical study of CW Raman amplification in silicon–nanocrystal waveguides, exploiting the giant Raman gain of silicon nanocrystals [28,29], was proposed. In [31], silicon nanocrystals embedded in a ~7 µm long slotted photonic crystal waveguide (PCWG) was designed, showing an SRS net gain of the order of 11 dB. Thereafter, in [32], the design of a silicon Raman amplifier based on silicon nanocrystals embedded in a 4 µm long slotted PCWG, yielding to an overall gain of ~3.22 dB at a bit rate of 400 Gbps, was reported. In [33], the design of an integrable all-silicon Raman laser of a footprint of 7μm, based on a slotted photonic crystal nanocavity, which exhibits a lasing efficiency of 18% at a wavelength of 1552 nm, with an optical threshold power of the order of 0.5 μW, was described. 

In [34], a strong SRS and very high Raman gain in optical cavities made of Si nanowire of various diameters in the visible region were reported. The enhancement of the Raman gain coefficient of Si nanowire was evaluated by a factor greater than 10^6^ at 532 nm excitation wavelength with respect to the gain value at the 1.55 µm wavelength reported in [28,29], even though the losses are estimated 10^8^ higher. 

Before concluding, we note that SRS in photonic devices not only enables Raman lasers for the generation of new frequencies but can also lead to nontrivial, nonlinear interactions through tailoring the dispersion properties, such as the interplay between the Raman effect, and both χ^(2)^ and χ^(3)^ effects, impacting Kerr comb formation, electro-optic comb formation and supercontinuum generation [22,35].

In conclusion, it is worth noting that while the implementation of microphotonics devices has been facilitated by the development of microscales fabrication techniques, the realization of nanodevices is still an issue. The big challenge of the future is a reduction in the size of integrated optical devices towards monolithically integrable, nanoscale low-powered Si Raman lasers, while maintaining a high level of performance. 
